# Treatment of thoracic actinomycosis: A retrospective analysis of 40 patients

**DOI:** 10.4103/1817-1737.62470

**Published:** 2010

**Authors:** Jae-Uk Song, Hye Yun Park, Kyeongman Jeon, Sang-Won Um, O Jung Kwon, Won-Jung Koh

**Affiliations:** *Department of Medicine, Division of Pulmonary and Critical Care Medicine, Samsung Medical Center, Sungkyunkwan University School of Medicine, Seoul, Korea*

**Keywords:** Actinomycosis, drug therapy, thoracic diseases, treatment outcomes

## Abstract

**BACKGROUND:**

The aim of this study was to evaluate treatment outcomes in patients with thoracic actinomycosis and identify patient characteristics associated with unfavorable responses to antibiotic therapy.

**METHODS:**

A retrospective analysis was performed on 40 patients with pathologically confirmed thoracic actinomycosis.

**RESULTS:**

Initial surgical treatment was performed on 17 patients to control severe symptoms such as hemoptysis or rule out lung cancer. Sixteen (94%) patients were successfully treated, including three patients who did not receive postoperative antibiotics, and one patient died of a postoperative complication. The median duration of oral antibiotic therapy after surgery was 3 months. After the diagnosis of actinomycosis, 23 patients began antibiotic therapy. The median duration of oral antibiotic therapy was 5 months. Favorable treatment outcomes were achieved in 18 of these 23 patients (78%), while five (22%) showed unfavorable responses to antibiotic therapy. Surgery was successfully performed in these five patients. The patients with unfavorable responses to antibiotic therapy had a longer duration of symptoms prior to treatment (median, 10 months) as compared to patients with favorable responses (median, 2 months; *P* = 0.012).

**CONCLUSIONS:**

Medical treatment failure is possible in patients with thoracic actinomycosis, and close monitoring is necessary in those who begin antibiotic therapy. In addition, surgical resection may be a valid option for patients who do not respond to antibiotic therapy, with the consideration of the age and comorbid conditions.

Thoracic actinomycosis is an uncommon indolent infection caused by anaerobic or microaerophilic bacteria, principally in the genus *Actinomyces*. Thoracic involvement results from aspiration of oropharyngeal or gastrointestinal secretions into the respiratory tract[1,2] and commonly presents as pulmonary infiltrate or a mass.[3]

With regard to the treatment of thoracic actinomycosis, intravenous (IV) antibiotics are administered for 2–6 weeks followed by a prolonged period of oral antibiotics.[4] Several recent studies, however, have reported successful cases of thoracic actinomycosis with relatively brief courses of antibiotic therapy.[5–10] Our previous data on the treatment of thoracic actinomycosis also demonstrate that oral antibiotic treatment with short-term IV antibiotics or without the need for IV antibiotics is feasible.[9] Favorable results from this short-term treatment may be explained by early diagnosis followed by antibiotic therapy and available modern imaging modalities for monitoring patient responses to treatment.[11]

Following our previous reports, however, we found that some patients showed persistent symptoms and progressive radiographic lesions despite continuous antibiotic treatment. Hence, the purpose of this study was to evaluate the treatment outcomes of patients with thoracic actinomycosis and to investigate patient characteristics associated with unfavorable responses to antibiotic treatment.

## Methods

### Patients

All 40 patients who were diagnosed with thoracic actinomycosis at the Samsung Medical Center (a 1250-bed referral hospital in Seoul, Korea) between January 2004 and December 2008 were included in the present study. All patients had pathological confirmation of *Actinomyces* infection based on histopathologic findings of sulfur granules, or Gomori methenamine silver stain-positive branching filamentous organisms. Specimens were obtained from a surgical biopsy, bronchoscopic biopsy, percutaneous fine needle aspiration, or core biopsy.

### Data collection

Medical records of all patients included in the study were reviewed. Records included information regarding age, gender, presenting symptoms, comorbidities, diagnostic methods, therapeutic modalities and the duration of IV or oral antibiotic treatment and clinical outcome. We also examined the initial and follow-up chest radiographs and chest computed tomography (CT) scans. Lymph nodes greater than 1 cm in transverse diameter were regarded as enlarged lymph nodes. Lung lesions were categorized as consolidation, nodule or mass, bronchiectasis, atelectasis and endobronchial lesions based on predominant features.[12]

### Treatment outcomes

During the follow-up period, attending physicians routinely checked medication compliance at every visit during the treatment period for all enrolled patients. Moreover, we regularly examined responses to treatment (e.g., clinical symptoms and chest radiographic findings) at 4, 8, 12, 16 and 20 weeks after the initiation of treatment.

A favorable outcome was defined as follows; improved clinical symptoms and disappearance of the main lesion on thoracic imaging and reduction to residual scarring. On the basis of these findings, attending physicians would decide whether to discontinue antibiotic therapy.[9,10] An unfavorable outcome was defined as surgical removal due to persistent clinical symptoms and the progression of the main lesion on thoracic imaging despite antibiotic therapy.

The Institutional Review Board of Samsung Medical Center approved the study protocol. Informed consent was waived due to the retrospective nature of the study.

### Statistical analysis

Because a majority of the data did not follow a normal distribution, all results presented here are expressed as the median and interquartile range (IQR, 25^th^ and 75^th^ percentiles) or the number (percentage) of patients. Categorical variables were analyzed using the Pearson χ^2^-test or Fisher's exact test. Continuous variables were analyzed using a Mann–Whitney *U*-test. All *P* values were two-sided, with *P* < 0.05 considered to be significant. Analyses were executed using PASW, version 17.0 for Windows (SPSS Inc., Chicago, IL).

## Results

### Patient characteristics

Patient characteristics are listed in Table 1. The median age was 55 years (IQR, 46–61 years); 28 males (70%) and 12 females (30%) were included in the study. A history of smoking was found in 25 patients, and 14 patients had underlying pulmonary comorbidities such as tuberculosis (*n* = 5), chronic obstructive pulmonary disease (*n* = 3), bronchiectasis (*n* = 3), pulmonary aspergilloma (*n* = 2) and lung cancer (*n* = 1).

**Table 1:** Baseline characteristics of 40 patients with thoracic actinomycosis

Variables	Number (%) or median (interquartile range)
Age, years	55 (46–61)
Gender, male	28 (70)
Nonsmoker	15 (38)
Pulmonary comorbidity	14 (35)
Tuberculosis or nontuberculous	
mycobacterial disease	5 (13)
Chronic obstructive pulmonary disease	3 (7)
Bronchiectasis	3 (7)
Pulmonary aspergilloma	2 (5)
Lung cancer	1 (3)
Symptoms	
Hemoptysis	24 (60)
Cough	22 (55)
Sputum	18 (45)
Chest pain	7 (18)
Fever	6 (15)
Duration of symptoms, months	3 (1–7)
Suspected diagnosis for hospitalization	
Lung cancer	18 (45)
Actinomycosis	7 (18)
Tuberculosis or nontuberculous	
mycobacterial disease	7 (18)
Pulmonary aspergilloma	3 (7)
Lung abscess/pneumonia	2 (5)
Other	3 (7)

The most common presenting symptoms were hemoptysis (*n* = 24) and cough (*n* = 22). Massive hemoptysis defined as the expectoration of greater than 200 ml of blood within a day[3] occurred in six patients. The median duration of symptoms was 3 months (IQR, 1–7 months). On the basis of clinical and radiological findings, the most common suspected diagnosis was lung cancer (*n* = 18), followed by tuberculosis or nontuberculous mycobacterial disease (*n* = 7).

### Radiographic features

Initial chest radiography and CT were available for all patients. The most common chest radiographic findings were consolidation and a mass or nodule [Table 2]. Most lesions showed right lobe predilection and nine patients had endobronchial lesions. Seven of these patients had also parenchymal lesions including consolidation (*n* = 4), a mass (*n* = 2) and atelectasis (*n* = 1). The remaining two patients had only endobronchial lesions.

**Table 2:** Radiological findings of a simple chest X-ray and CT (n= 40)

Variables	Number (%) or median (interquartile range)
Site of lesions	
Parenchymal lesions, right/ left	23/15 (58/37)
Endobronchial lesions, right/ left	8/1 (21/2)
Chest X-ray findings	
Consolidation	23 (57)
Mass or nodule	12 (30)
Atelectasis	2 (5)
Bronchiectasis	1 (2)
Nonparenchymal lesion	2 (5)
Chest CT findings	
Size of parenchymal lesion, cm (*n* = 37)*	6.1 (3.9-7.7)
Low attenuation within the main lesion	21 (53)
Mediastinal or hilar lymph node enlargement	19 (48)
Endobronchial lesion	9 (22)
Cavitation	8 (20)
Pleural invasion	8 (20)

* Data for two patients with endobronchial lesions alone and one patient with bronchiectasis and bronchiolitis in the left lower lobe were not available to estimate the size of the lesion

Lung parenchymal lesions had a median size of 6.1 cm (IQR, 3.9–7.7 cm) and the low-attenuation core within the consolidation or mass was present in 21 patients. Mediastinal or hilar lymph node enlargement was observed in 19 patients and pleura invasion was present in eight patients; however, severe complications such as empyema, chest wall sinus fistula, pericarditis, or mediastinitis were not detected in our study.

### Diagnosis and treatment

The confirmatory diagnosis was performed by a surgical lung biopsy (*n* = 18), percutaneous fine needle aspiration (*n* = 10), core biopsy (*n* = 7), bronchoscopic biopsy (*n* = 4) and transbronchial lung biopsy (*n* = 1). The indications for surgery were severe symptoms such as continuous or massive hemoptysis (*n* = 10) and consideration of probability of lung cancer (*n* = 6). Two patients initially received empirical antibiotic therapy under presumptive diagnosis of thoracic actinomycosis and surgery was performed after observation of unfavorable responses.

In 23 patients, treatment was initiated with antibiotics after diagnosis of thoracic actinomycosis. Intravenous or oral amoxicillin–clavulanate (amoxicillin 1500–1750 mg and potassium clavulanate 250–375 mg/day) was used on the basis of previous studies.[3,9,13] The median duration of total antibiotic treatment including both IV and oral antibiotics was 5.1 months (IQR, 4.0–6.1 months) and the duration of IV antibiotic treatment ranged from 0 to 14 days (median, 0 day; IQR, 0–2 days). Only one patient with large amounts of pleural effusion received IV antibiotics for 14 days, and a pigtail catheter for pleural effusion drainage was inserted. The median duration of oral antibiotic therapy was 5.0 months (IQR, 4.0–6.0 months). Fifteen patients received oral antibiotic therapy without IV antibiotic therapy. Favorable outcomes were obtained at the end of treatment in 18 of the 23 patients (78%). Unfavorable responses to antibiotic therapy were observed in five patients. These five patients eventually underwent surgical lobectomy for control of actinomycosis at 3 months (*n* = 1), 5 months (*n* = 3) or 8 months (*n* = 1) from the initiation of antibiotic treatment. All of these patients showed favorable outcomes after surgery without postoperative complications.

Surgery followed by antibiotic therapy was performed on 17 patients. Two patients underwent segmentectomy and 14 patients received a lobectomy. One patient underwent bronchoscopic removal for broncholithiasis. The median duration of IV antibiotic treatment was 7 days (IQR, 4–12 days). One patient suffered postoperative bronchoesophageal fistula and empyema, and died at 170 days after lobectomy. The median duration of oral antibiotic therapy in the remaining patients was 3.0 months (IQR, 1.0–4.5 months). Three of 17 patients did not receive oral antibiotic treatment after the operation and eight patients received less than 3 months of postoperative oral antibiotics. A favorable outcome was obtained at the end of antibiotic treatment in 16 patients including three patients without postoperative antibiotic therapy. However, one patient died due to postoperative complications.

### Comparisons between patients with surgical treatment and those without surgical treatment

The comparison between patients with surgical treatment and those without surgical treatment is summarized in Table 3. The patients without surgical treatment were more likely to have underlying pulmonary comorbid disease. The frequency of massive hemoptysis was higher in patients with surgical treatment; however, this was not statistically significant. The duration of antibiotic therapy in patients with surgical treatment was significantly shorter compared to that in patients without surgical treatment. However, there was no significant difference in treatment outcomes.

**Table 3:** Comparisons between patients without initial surgery and those with initial surgery

Variables	Patients with surgical treatment* (*n* = 17)	Patients without surgical treatment (*n* = 23)	*P* value
Age, years	52 (45–57)	57 (47–65)	0.242
Gender, male	8 (47)	20 (87)	0.006
Pulmonary comorbid condition	10 (59)	4 (17)	0.007
Smoking history	8 (47)	18 (78)	0.041
Massive hemoptysis	5 (29)	1 (4)	0.067
Duration of symptoms, months	3.0(1.0–6.0)	2.0 (1.0–4.8)	0.935
CT characteristics of the lesion			
Consolidation feature	8 (47)	14 (61)	0.385
Size of the parenchymal lesion, cm	5.5 (3.7–7.7)	6.4 (3.7–7.6)	0.703
Low attenuation core within the lesion	4 (24)	17 (74)	0.002
Size of the low attenuation core, cm	2.4 (1.9–3.2)	2.5 (1.8–4.3)	0.829
Endobronchial lesion	4 (24)	5 (22)	1.000
Pleural invasion	1 (6)	7 (30)	0.107
Duration of antibiotic treatment			
Duration of IV antibiotics, days	7 (4–12)	0 (0–2)	<0.001
Duration of oral antibiotics, months	3.0 (1.0–4.5)	5.0 (4.0–6.0)	0.002
Total duration of antibiotics, months	3.1 (1.1–5.8)	5.1 (4.0–6.1)	0.048
Favorable outcomes	16 (94)	18 (78)	0.216

Data are presented as the median (interquartile range) or number (%);

* One out of 17 patients underwent initial bronchoscopic removal for endobronchial actinomycosis.

### Comparisons between patients with favorable responses and those with unfavorable responses to antibiotic therapy

Comparisons of the clinical and radiographic characteristics and treatment duration between patients with favorable responses and those with unfavorable responses to antibiotic therapy are summarized in Table 4. No significant differences were present in age, gender, pulmonary comorbid condition, smoking history or the presence of massive hemoptysis. Additionally, no significant differences were observed in the location and size of the lesion, the presence of consolidation, the presence and size of central low attenuation and endobronchial lesion and the presence of pleura invasion between the two groups. The duration of antibiotic treatment did not differ, but patients with an unfavorable response to antibiotic therapy had a longer duration of symptoms before diagnosis (median, 10.0 months; IQR, 3.5–14.5 months) as compared to patients with favorable responses to antibiotic therapy (median, 2.0 months; IQR, 1.0–4.8 months; *P* = 0.012).

**Table 4:** Comparisons between patients with favorable responses and those with unfavorable responses to antibiotic therapy

Variables	Favorable outcome (n = 18)	Unfavorable outcome (n = 5)	*P* value
Age, years	58 (50–65)	46 (42–58)	0.067
Gender, male	16 (89)	4 (80)	0.539
Pulmonary comorbid condition	4 (22)	0 (0)	0.539
Smoking history	15 (83)	3 (60)	0.291
Massive hemoptysis	1 (6)	0 (0)	0.590
Duration of symptoms, months	2.0 (1.0–4.8)	10.0 (3.5–14.5)	0.012
CT characteristics of the lesion			
Consolidation feature	9 (50)	5 (100)	0.116
Size of the parenchymal lesion, cm	5.7(2.2–7.3)	6.7(6.0–11.5)	0.164
Low attenuation core within the lesion	12 (67)	5 (100)	0.272
Size of the low attenuation core, cm	2.2 (1.3–4.2)	3.1 (2.4–4.5)	0.328
Endobronchial lesion	3 (17)	2 (40)	0.291
Pleural invasion	5 (28)	2 (40)	0.621
Duration of antibiotic treatment			
Duration of IV antibiotics, days	0 (0–3)	1 (0–2)	0.638
Duration of oral antibiotics, months	5.2 (3.8–6.0)	5.0 (4.0–7.0)	0.971
Total duration of antibiotics, months	5.4 (3.8–6.1)	5.1 (4.0–7.0)	0.914
Response during follow-up period*			
4 weeks after receiving antibiotics	15 (83)	1 (20)	0.017
8 weeks after receiving antibiotics	18 (100)	2 (40)	0.006
12 weeks after receiving antibiotics	18 (100)	0 (0)	<0.001
16 weeks after receiving antibiotics	18 (100)	0 (0)	<0.001
20 weeks after receiving antibiotics	18 (100)	0 (0)	<0.001

Data are presented as the median (interquartile range) or number (%).

* Data for two patients were unavailable to evaluate their responses to antibiotics due to insufficient data collection within 4 weeks after starting treatment

During the follow-up period, patients with favorable responses showed subsequent resolution after 8 weeks of treatment. In contrast, patients with unfavorable responses did not show improvements with antibiotic therapy after 12 weeks from the initiation of treatment.

## Discussion

In the present study, we found that all patients receiving surgical removal followed by short-term antibiotic treatment had successful treatment outcomes, except for one patient with postoperative complications, while 78% (18/23) of patients who received antibiotic therapy alone were successfully treated. Many patients (22%5/23) were refractory to antibiotic therapy and eventually underwent surgical resection.

Diagnosis of thoracic actinomycosis is usually hampered by difficulty in isolating *Actinomyces*. In our study, positive cultures were obtained in only three out of 40 patients, and this low yield of sputum or bronchial washing fluid culture was consistent with previous reports.[14–16] In addition, the cultures through sputum or bronchial washing fluid are inadequate for the diagnosis of thoracic actinomycosis, because Actinomycosis is normal inhabitant of the oropharynx.[14,15] The culture result of *Actinomyces odontolyticus* obtained in our three patients was also a part of indigenous oral flora in humans and a less commonly recognized pathogen of the *Actinomycoses* group. Therefore, the diagnosis of thoracic actinomycosis in present study was made based on histopathologic findings.

Actinomycosis has been traditionally thought of as a medically treatable disease with good prognosis.[14] The main treatment principle in thoracic actinomycosis is to use long-duration intensive antibiotic treatment. The need for this intensive treatment is presumably due to the drug's poor penetration, which is caused by avascularity and dense co-aggregations of actinomyces known as sulphur granules.[4,15] Since current presentations of thoracic actinomycosis tend to be milder than in past reports and imaging modalities that monitor responses to treatment are available, short-term treatment regimens may obtain successful outcomes during thoracic actinomycosis treatment.[5–10] With respect to short-term treatment outcomes in thoracic actinomycosis, Kolditz *et al.* recently reported that the sustained cure rate of antibiotic treatment was 85%; moreover, in the remaining 15% of patients, the main lesion, which had resolved during a less than 3-month antibiotic treatment, recurred after the treatment was stopped.[10] Furthermore, a previous study performed in our center demonstrated that traditional recommendations of IV antibiotic therapy for 2–6 weeks followed by oral antibiotic therapy for 6–12 months was not necessary for all patients, and the main lesions of thoracic actinomycosis were subsequently resolved.[9]

However, during the regular monitoring of therapeutic responses based on chest images, the present study found that five of 23 patients (22%) who received medical antibiotic treatment did not show improvements in the main lesions or clinical symptoms despite more than 3 months of antibiotic therapy. When clinical and radiographic features of those with favorable and unfavorable outcomes were compared to determine why medical therapy was not effective in some patients, the only significant difference was the duration of symptoms prior to treatment. This result is consistent with a report by Slade *et al.*, which suggested that the response to therapy is determined by the chronicity of the infection.[16]

Another possible explanation for the unfavorable responses to antibiotic therapy may be the duration of the initial IV antibiotic therapy. Specifically, the median duration of IV antibiotic treatment used in our study was shorter (0 day; range, 0–14 days) as compared to other studies that used a median duration of over 2 weeks.[6–8,10,17] Moreover, two of five patients with unfavorable outcomes did not received IV antibiotics. This duration of IV antibiotic treatment, however, was similar to that of our previous study,[9] suggesting that oral antibiotic treatment without IV antibiotics or a shorter duration treatment of IV antibiotics may be adequate for treating thoracic actinomycosis in some patients. Additionally, the duration of IV antibiotic treatment was not significantly different between patients with favorable responses and those with unfavorable responses to antibiotic therapy. Therefore, whether the duration of IV antibiotics affected treatment outcomes in the present study is not clear.

Finally, the antibiotic resistance might be considered as a cause of unfavorable outcome as *Actinomyces* strain. According to the previous report,[12] *Actinomyces* species are susceptible to a wide range of β-lactam agents, and these, when combined with β-lactamase inhibitors, are regarded as agents of first choice. In the present study, five patients showed unfavorable responses after antibiotic therapy, although they received antibiotics of amoxicillin–clavulanate. Because diagnoses in these five patients were made based only on histological results without culture, antimicrobials susceptibility assays could not be performed. This is a limitation of this retrospective study.

According to previous reports, diminution in the shadowing on a chest radiograph is expected within 4 weeks.[4] Our study found that within 4 weeks, 83% (15/18) of patients with favorable responses to antibiotic therapy demonstrated improvements in the main lesion, while only 20% (1/5) of patients with unfavorable responses showed any response to antibiotic therapy. Specifically, patients with unfavorable outcomes had not responded after 12 weeks of initiating antibiotic treatment. Therefore, based on our results, early antibiotic responses may be an important clue in determining the cause of medical treatment failure, even though the optimal time to respond to antibiotics is not clear.

Due to the satisfactory success rate of antibiotic treatment, the role of surgery has been often controversial.[18] Surgery for actinomycosis has been suggested as an important adjunctive option for those with recurrent or life-threatening hemoptysis, an uncertain diagnosis of cancer, or who may need complication management. Our results showed that surgical resection was effective in all patients who received surgical intervention in conjunction with an antibiotics treatment to rule out the diagnosis of cancer, excluding one patient with postoperative complications, and all five patients with unfavorable responses to antibiotic treatment. The only patient who died of postoperative complications was 81-year-old male with several comorbidities such as chronic obstructive pulmonary disease, bronchiectasis and poorly controlled diabetes mellitus. He received left lower lobectomy to control massive hemoptysis. During postoperative care, bronchoesophageal fistula and empyema developed and he died of these complications. Therefore, if the age and comorbid conditions were considered, we suggest that surgery may be a valid option for treating thoracic actinomycosis in patients who show unfavorable responses to antibiotic treatment.

The present study has several limitations. First, it was a non-randomized, retrospective study performed at a single center over a five-year period, which could have led to selection bias. In addition, due to the absence of a consistent policy for deciding when to perform surgery, the timing of surgery varied by attending physician. In addition, the present study was conducted on a small sample of patients. Therefore, the sample size might have influenced the statistical power and thus limit the generalization of the findings.

In conclusion, medical treatment failure can occur in patients with thoracic actinomycosis, and close monitoring is necessary in those who begin antibiotic therapy. Furthermore, surgical resection may be considered in patients who do not respond to antibiotic therapy, with the consideration of the age and comorbid conditions.
